# Reference Gene Selection for Quantitative Real-time PCR Normalization in *Caragana intermedia* under Different Abiotic Stress Conditions

**DOI:** 10.1371/journal.pone.0053196

**Published:** 2013-01-02

**Authors:** Jianfeng Zhu, Lifeng Zhang, Wanfeng Li, Suying Han, Wenhua Yang, Liwang Qi

**Affiliations:** 1 Laboratory of Cell Biology, Research Institute of Forestry, Chinese Academy of Forestry, Beijing, China; 2 Key Laboratory of Research Institute of Forest Ecology and Protection, Chinese Academy of Forestry, Beijing, China; University of New England, Australia

## Abstract

Quantitative real-time reverse transcription polymerase chain reaction (qPCR), a sensitive technique for gene expression analysis, depends on the stability of the reference genes used for data normalization. *Caragana intermedia*, a native desert shrub with strong drought-resistance, sand-fixing capacity and high forage value that is widespread in the desert land of west and northwest China, has not been investigated regarding the identification of reference genes suitable for the normalization of qPCR data. In this study, 10 candidate reference genes were analyzed in *C. intermedia* subjected to different abiotic (osmotic, salt, cold and heat) stresses, in two distinct plant organs (roots and leaves). The expression stability of these genes was assessed using geNorm, NormFinder and BestKeeper algorithms. The best-ranked reference genes differed across the different sets of samples, but *UNK2*, *PP2A* and *SAND* were the most stable across all tested samples. *UNK2* and *SAND* would be appropriate for normalizing gene expression data for salt-treated roots, whereas the combination of *UNK2*, *SAND* and *EF-1α* would be appropriate for salt-treated leaves. *UNK1*, *UNK2* and *PP2A* would be appropriate for PEG-treated (osmotic) roots, whereas the combination of *TIP41* and *PP2A* was the most suitable for PEG-treated leaves. *SAND*, *PP2A* and *TIP41* exhibited the most stable expression in heat-treated leaves. In cold-treated leaves, *SAND* and *EF-1α* were the most stably expressed. To further validate the suitability of the reference genes identified in this study, the expression levels of *DREB1* and *DREB2* (homologs of *AtDREB1* and *AtDREB2*) were studied in parallel. This study is the first systematic analysis for the selection of superior reference genes for qPCR in *C. intermedia* under different abiotic stress conditions, and will benefit future studies on gene expression in *C. intermedia* and other species of the leguminous genus *Caragana*.

## Introduction

Quantitative real-time reverse transcription polymerase chain reaction (qPCR) is an efficient, specific, and reproducible method for quantifying transcript expression levels, and is widely used to analyze mRNA in different organisms [1], developmental stages [2], [3] and responses to abiotic and biotic stress [4]–[7]. However, the accuracy of qPCR is influenced by a number of variables, such as RNA stability, quantity, purity, enzymatic efficiency in cDNA synthesis and PCR amplification [8]. Thus, to avoid bias, a normalization step is an essential pre-requisite. The most accepted approach for normalization is to include one or a small number of reference genes (internal control genes), whose expression is presumed stable in control and experimental conditions [9], [10].

The traditional reference genes are mostly cellular maintenance genes, such as 18S ribosomal RNA (*18S rRNA*), actin (*ACT*), tubulin (*TUB*), glyceraldehyde-3-phosphate dehydrogenase (*GAPDH*), and elongation factor 1-α (*EF1-α*) [11], [12]. However, recent studies indicate that these genes are not always stably expressed when tested in other species or under a wider range of experimental treatments [13]–[15]. Recently, some new reference genes were identified by microarray analyses in *Arabidopsis thaliana* and soybean that show highly stable expression levels [16], [17]. These reference genes include SAND family protein (*SAND*), protein phosphatase 2A (*PP2A*), TIP41-like family protein (*TIP41*), F-box/kelch-repeat protein (*F-box*), phosphoenolpyruvate carboxylase-related kinase 1 (*PEPKR1*) and others. Many of these reference genes were found to outperform traditional reference genes, for example, *PP2A* in hybrid roses [18], *SAND* in buckwheat [19] and *TIP41* in peanut [20] were the most stably expressed genes in those systems. Therefore, systematic validation of reference genes is essential for certain experimental conditions and in different species [10]. Statistical algorithms, such as geNorm [21], NormFinder [22], and BestKeeper [23], have been used to identify the best reference genes for qPCR data normalization under different experimental conditions.

To date, studies of reference gene expression in plants have mainly focused on model and important crop species, such as *Arabidopsis* [16], rice [24], poplar [25], soybean [15], [17], wheat [26], barley [27], tomato [13], *Vitis* [28], and *Medicago truncatula* [29]. However, no systematic analysis for the selection of reference genes for qPCR in *Caragana intermedia* has been found.

*C. intermedia* belongs to the family Fabaceae, and is a native desert shrub with strong drought-resistance, sand-fixing capacity and high forage value that is widespread in the desert land of west and northwest China [30]. From a scientific standpoint, it has proven an ideal material for studying the mechanisms of drought and salt tolerance of shrubs in China, because of its easy cultivation and strong abiotic resistance [30]–[32].

In this study, 10 candidate reference genes (*ACT7*, *TUA5*, *EF*-*1*α, *PP2A*, *SAND*, *TIP41*, *F-box*, *PEPKR1*, *UNK1*, *UNK2*) were selected because of their stable expression in microarray studies in *A. thaliana* and soybean [16], [17]. The stability of these genes was analyzed in *C. intermedia* subjected to different abiotic (osmotic, salt, cold and heat) stresses, in two distinct plant organs (roots and leaves). Furthermore, to validate the selection of candidate reference genes, the expression levels of *DREB1* and *DREB2* homologues were assessed using different reference genes. This work will benefit future studies on gene expression in *C. intermedia* and other species of the leguminous genus *Caragana*.

## Results

### Expression Profiling of Candidate Reference Genes

**Figure 1:**
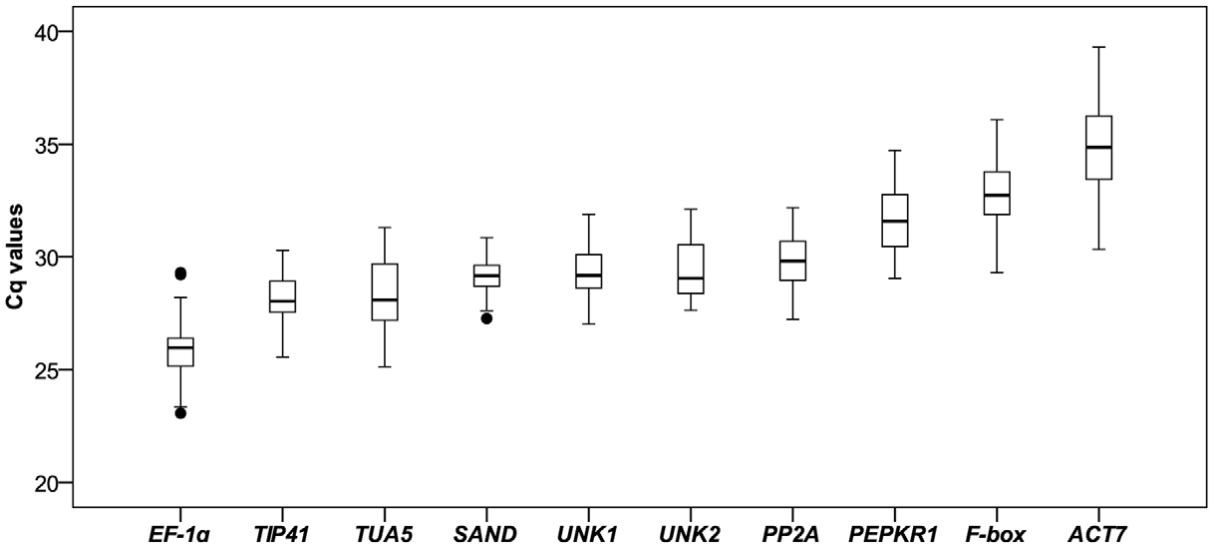
Expression levels of candidate reference genes across all samples. Lines across the boxes depict the medians. Boxes indicate the interquartile range. Whiskers represent 95% confidence intervals, black dots represent outliers.

A total of 10 candidate reference genes were assessed using qPCR to quantify their mRNA levels (Table 1). The expression levels of the candidate reference genes were determined as quantification cycle (Cq) values, and the transcripts of these genes showed different levels of abundance (Figure 1). The mean Cq values of the genes ranged from 26–35, with most lying between 28 and 30 across all tested samples. *EF-1α* had the lowest Cq (mean Cq of 25.8), indicating the highest level of expression, *SAND*, *PP2A*, *TIP41*, *TUA5*, *UNK1* and *UNK2* were moderately expressed, *F-box* and *ACT7* were expressed at low levels (mean Cq of 32.9 and 34.9). *SAND* showed the least gene expression variation (coefficient of variation, CV, of 3.05%), while *ACT7* (5.78%) and *TUA5* (5.74%) were the most variable across all samples.

**Table 1:** *Caragana intermedia* candidate reference genes descriptions and comparison with *Arabidopsis* orthologs.

Gene symbol	Gene name	GenBankAccession	*Arabidopsis*homolog locus	Primer sequence (5′–3′)	Ampliconlength (bp)	Tm(°C)	PCRefficiency (%)
*ACT7*	Actin7	JX272637	AT5G09810	CCGAAGAGCATCCAGTTTTG	58	78.5	100.67
				TCACGGTTAGCCTTGGGGTT			
*EF-1α*	Elongation factor -1α	JX272638	AT5G60390	AGATGGTTCCCACTAAGCCTATG	115	82.6	93.13
				ACACTCTTGATGACTCCAACTGC			
*TUA5*	Alpha Tubulin	JX272639	AT5G19780	CTGATGTGGTTGTGCTTTTGGAC	183	80.8	93.11
				GGTTTGTCTGGAACTCGGTAATG			
*F-box*	F-box/kelch-repeat protein	JX272640	AT5G15710	AATGGGTCGTGGAGGGTCTA	71	82.3	94.80
				AACCCCTTGGCTTGGTCTTA			
*PEPKR1*	Phosphoenolpyruvate carboxylase-related Kinase 1	JX272641	AT1G12580	GAACAGTTGGGTTGGGGACA	181	82.4	94.05
				GATCCACAACATTCGGGTGC			
*PP2A*	Protein phosphatase 2A	JX272642	AT1G10430	TTTCGGATAGGAGGAAATGCAC	106	80.3	94.18
				TCAAGGCCACCAAAAGCGTA			
*SAND*	SAND family protein	JX272643	AT2G28390	ATACTCGTCAACAGCAGAAA	133	79.8	91.46
				GTCACCCAACATAAAAGAAC			
*TIP41*	TIP41-like family protein	JX272644	AT4G34270	CGTCCAAGAGTGGGAACAGA	167	81.7	90.22
				GAACTTCAACAGGCGGCAAG			
*UNK1*	Hypothetical protein	JX272645	AT1G31300	CAATGTTGAGTGGGGAGGGA	162	81.2	97.41
				CAACCAGCCAAGCAAGGAAT			
*UNK2*	Hypothetical protein	JX272646	AT4G33380	CAAAGATAGTGCTGCTGATTGC	147	81.6	92.64
				TCCTGGTGTTTGTGCTGATAGA			
DREB1^*^	Dehydration responsive element binding protein 1	KC123242	AT4G25480	TTCTGACCCACAACCTTACTC	167	86.5	96.40
				TCTTCTTGTTTGGTTCCCTTA			
DREB2^*^	Dehydration responsive element binding protein 2	JX272647	AT2G40340	GAAAGGGTGTATGAAAGGTA	228	83.4	92.56
				GTTATGTGAGGGAAGTTGAG			

Notes: * used for normalization validation under cold or salt stress conditions.

**Figure 2:**
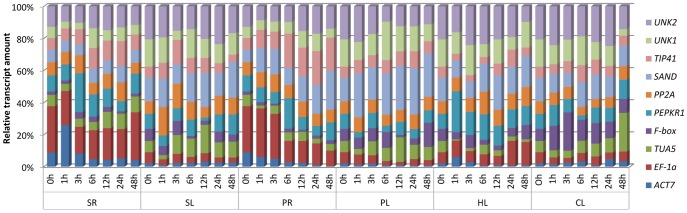
Distribution of relative transcript amount of the reference genes across all samples. Transcript amount are represented as percentages of the aggregated 10-transcript pool for each sample. SR (0–48 h), roots exposed to high-salt; SL (0–48 h), leaves exposed to high-salt treatment; PR (0–48 h), roots exposed to PEG treatment; PL (0–48 h), leaves exposed to PEG treatment; HL (0–48 h), leaves exposed to heat treatment; CL (0–48 h), leaves exposed to cold treatment.

The variation in relative transcript amount of the reference genes across all tested samples is shown in Figure 2. Transcript amounts are represented as percentages, relative to the aggregated reference transcript pool for each sample. The proportions of *PP2A*, *SAND* and *UNK2* transcript remained relatively constant, while those of *ACT7*, *TUA5* and *F-box* were more variable across all samples. The transcript levels of *UNK1* remained relatively constant in PEG-treated roots (PR) or leaves (PL), and those of *TIP41* were also relatively constant in the PL and cold-treated leaves (CL). Although the expression level of *EF-1α* was more variable across all samples and particularly high in the PR and salt-treated roots (SR), its expression was relatively constant in the CL and salt-treated leaves (SL). These results clearly suggested that the expression level of none of the reference genes is truly constant, and varies in different spatial and temporal patterns and environmental conditions.

### Expression Stability of Candidate Reference Genes

#### a) geNorm analysis

The expression stability of the 10 reference genes was assessed using the geNorm software. The geNorm algorithm is based on the principle that the logarithmically transformed expression ratio between two genes should be constant if both genes are stably expressed in a given sample set. The candidate reference genes were ranked by geNorm based on the expression stability value M, which is calculated for all genes being investigated (the lower the M value, the higher the gene's expression stability) [21].

**Figure 3:**
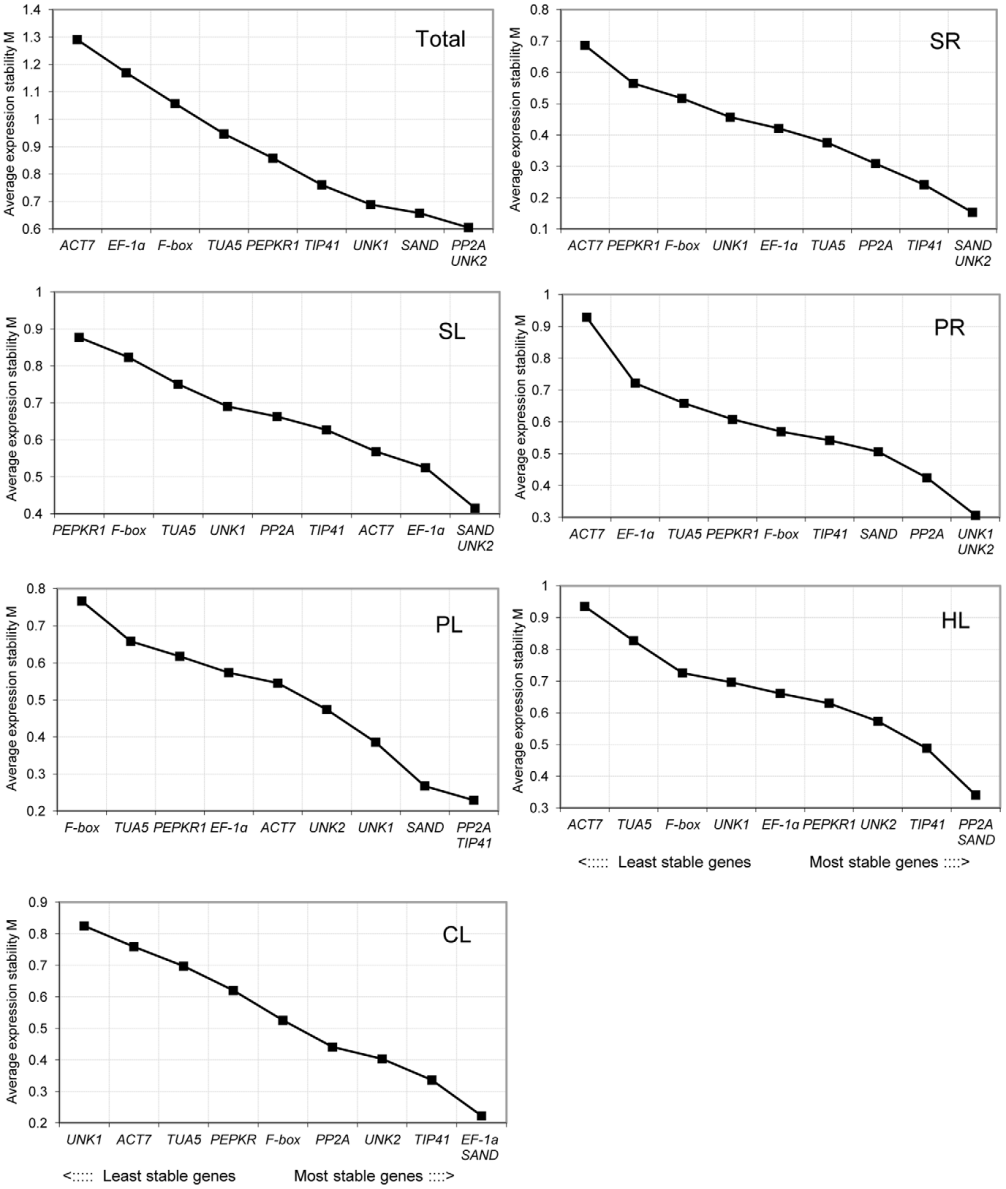
Gene expression stability and ranking of 10 reference genes as calculated by geNorm. Mean expression stability (M) was calculated following stepwise exclusion of the least stable gene across all treatment groups. The least stable genes are on the left, and the most stable on the right.

Figure 3 shows the ranking of the tested genes according to their expression stability in the *C. intermedia* samples, using data from six sets of treatment. When all 38 samples were analyzed together, *UNK2*, *PP2A*, and *SAND* were the most stable genes, while *ACT7* and *EF-1α* were the least stable (in order). In salt stress treatments, *UNK2* and *SAND* were the most stable genes, while *ACT7* and *PEPKR1* were the least stable in the SR and SL treatments, respectively. In the PR treatment, *UNK2* and *UNK1* were the most stable genes and *ACT7* the least; In the PL treatment, *TIP41* and *PP2A* were the most stable and *F-box* the least. In heat-treated leaves (HL), *SAND* and *PP2A* were the most stable genes and *ACT7* was the least stably expressed. In the CL treatment, the expression levels of *SAND* and *EF-1α* were the most stable and *UNK1* was the least stable. In addition, all of the tested reference genes showed relatively high stability with M values of less than 1.5, which is below the default limit of M ≤1.5.

**Figure 4:**
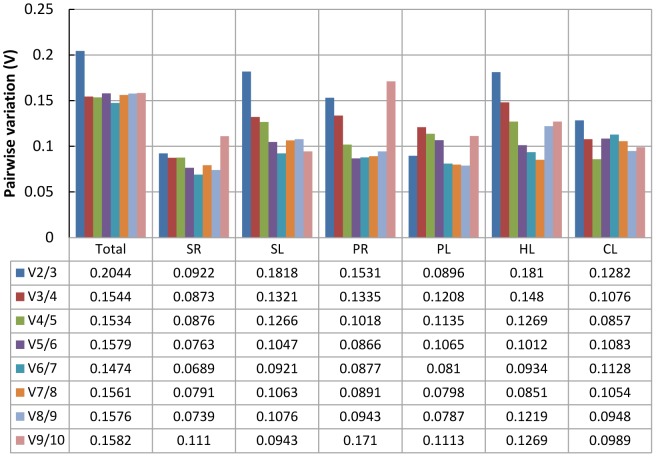
Determination of the optimal number of reference genes required for effective normalization. Pairwise variation (V_n/Vn +1_) analysis between the normalization factors (NF_n_ and NF_n +1_) was performed by the geNorm program to determine the optimal number of reference genes, and carried out for qPCR data normalization in various sample pools.

geNorm performs a stepwise calculation of the pairwise variation (V_n_/V_n+1_) between sequential normalization factors (NF_n_ and NF_n+1_) to determine the optimal number of reference genes required for accurate normalization. A large variation means that the added gene had a significant effect and should preferably be included for calculation of a reliable normalization factor [21]. As shown in Figure 4, the inclusion of a fourth gene had no significant effect (that is, low V_3/4_ value) for all pooled samples or for the SL treatment, so three reference genes would be optimal for normalizing gene expression under those conditions. Similarly, two reference genes would be sufficient for the SR and PL treatments, four for the PR and CL treatments, and five for the HL treatment. In the practical application, three reference genes for the PR (V_3/4_ = 0.1335) and HL (V_3/4_ = 0.1480) treatments, and two for the CL treatment (V_2/3_ = 0.1282) could also be accepted using a threshold value of 0.15 [14], [21], [33], [34].

#### b) NormFinder analysis

NormFinder program is a Visual Basic application tool for Microsoft Excel used to determine the expression stabilities of reference genes that ranks all reference gene candidates based on intra- and inter-group variations and combines both results into a stability value for each candidate reference gene [35]. The results of NormFinder analysis were slightly different from those of geNorm (Table 2). Both methods of analysis ranked as most stable *UNK2* and *SAND* in the SR treatment; *UNK2*, *SAND* and *EF-1α* in the SL treatment; *PP2A* and *TIP41* in the PL treatment; *PP2A*, *UNK1* and *UNK2* in the PR treatment. However, in the CL treatment, *PP2A* and *UNK2* emerged as the most stably expressed, whereas they were ranked fifth and fourth, respectively, by geNorm. In the HL treatment, *PP2A*, *UNK2* and *SAND* were in the top positions, while geNorm ranked *UNK2* in the fourth position. When evaluated across all experimental samples, *UNK2*, *PP2A* and *TIP41* were in the top positions, whereas *TIP41* was ranked fifth by geNorm.

**Table 2:** Expression stability of the reference genes calculated by NormFinder software.

Rank	Total		SR		SL		PR		PL		HL		CL	
	Gene	Stability	Gene	Stability	Gene	Stability	Gene	Stability	Gene	Stability	Gene	Stability	Gene	Stability
1	*PP2A*	0.188	*UNK2*	0.060	*UNK2*	0.163	*PP2A*	0.157	*PP2A*	0.079	*PP2A*	0.238	*PP2A*	0.142
2	*UNK2*	0.268	*SAND*	0.066	*SAND*	0.268	*UNK1*	0.234	*TIP41*	0.200	*UNK2*	0.303	*UNK2*	0.154
3	*TIP41*	0.462	*PP2A*	0.186	*EF-1α*	0.283	*UNK2*	0.301	*UNK2*	0.268	*SAND*	0.306	*SAND*	0.160
4	*SAND*	0.465	*TIP41*	0.255	*ACT7*	0.356	*F-box*	0.304	*UNK1*	0.316	*UNK1*	0.326	*TIP41*	0.197
5	*UNK1*	0.514	*EF-1α*	0.262	*PP2A*	0.368	*PEPKR1*	0.358	*SAND*	0.332	*PEPKR1*	0.360	*EF-1α*	0.261
6	*PEPKR1*	0.531	*F-box*	0.348	*TIP41*	0.415	*EF-1α*	0.434	*ACT7*	0.353	*TIP41*	0.369	*F-box*	0.439
7	*TUA5*	0.700	*TUA5*	0.376	*UNK1*	0.454	*TUA5*	0.435	*EF-1α*	0.386	*EF-1α*	0.422	*PEPKR1*	0.568
8	*F-box*	0.898	*UNK1*	0.387	*TUA5*	0.625	*SAND*	0.473	*PEPKR1*	0.407	*F-box*	0.435	*ACT7*	0.580
9	*EF-1α*	0.930	*PEPKR1*	0.427	*F-box*	0.628	*TIP41*	0.505	*TUA5*	0.519	*TUA5*	0.797	*TUA5*	0.597
10	*ACT7*	1.078	*ACT7*	0.763	*PEPKR1*	0.639	*ACT7*	1.179	*F-box*	0.765	*ACT7*	0.868	*UNK1*	0.673

Notes: SR, roots exposed to high-salt treatment; SL, leaves exposed to high-salt treatment; PR, roots exposed to PEG treatment; PL, leaves exposed to PEG treatment; HL, leaves exposed to heat treatment; CL, leaves exposed to cold treatment.

#### c) BestKeeper analysis

BestKeeper determines the most stably expressed genes based on the coefficient of correlation to the BestKeeper Index, which is the geometric mean of the candidate reference gene Cq values. BestKeeper also calculates the standard deviation (SD) and the coefficient of variation (CV) based on the Cq values of all candidate reference genes [23]. Genes with SD greater than 1 are considered unacceptable [36]. Reference genes are identified as the most stable genes, i.e. those that exhibit the lowest coefficient of variance and standard deviation (CV ± SD) [37]. The results of BestKeeper analysis are shown in Table 3. In the PL and CL treatments, the same four genes were identified by both the BestKeeper and geNorm programs, although their rank order was slightly altered. In the SL treatment, *UNK1* emerged as the most stably expressed (ranked seventh by geNorm and NormFinder). In the SR treatment, the same three genes, *UNK2*, *SAND* and *TIP41*, were identified by both the BestKeeper and geNorm programs, although their rank order was slightly altered. In the HL treatment, *EF-1α* emerged as the most stably expressed (ranked sixth by geNorm and seventh by NormFinder). When evaluated across all experimental samples, *SAND*, *TIP41* and *PP2A* were in the top positions, whereas *TIP41* was ranked fifth by geNorm and third by NormFinder.

**Table 3:** Expression stability of the reference genes calculated by BestKeeper software.

Rank	Total	SR	SL	PR	PL	HL	CL
1	*SAND*	*SAND*	*UNK1*	*SAND*	*SAND*	*EF-1α*	*SAND*
CV±SD	2.22±0.65	0.68±0.19	1.48±0.43	1.32±0.37	2.01±0.57	1.25±0.30	0.97±0.27
2	*TIP41*	*TIP41*	*UNK2*	*TIP41*	*TIP41*	*TIP41*	*TIP41*
CV±SD	2.89±0.81	0.74±0.20	1.57±0.40	1.54±0.45	2.23±0.62	1.54±0.39	1.22±0.34
3	*PP2A*	*UNK2*	*SAND*	*UNK1*	*UNK1*	*F-box*	*EF-1α*
CV±SD	3.22±0.96	0.90±0.26	2.02±0.56	1.97±0.60	2.35±0.68	1.66±0.56	1.40±0.34
4	*UNK1*	*UNK1*	*EF-1α*	*UNK2*	*PP2A*	*SAND*	*UNK2*
CV±SD	3.23±0.95	0.95±0.27	2.14±0.51	2.08±0.60	2.41±0.69	1.86±0.53	1.80±0.47
5	*UNK2*	*TUA5*	*F-box*	*PP2A*	*UNK2*	*PP2A*	*UNK1*
CV±SD	3.58±1.05	1.14±0.32	2.20±0.73	2.13±0.60	3.05±0.85	2.05±0.57	1.80±0.49
6	*PEPKR1*	*PP2A*	*PP2A*	*F-box*	*EF-1α*	*UNK1*	*F-box*
CV±SD	3.74±1.18	1.34±0.38	2.50±0.67	2.16±0.73	3.11±0.86	2.57±0.74	2.11±0.71
7	*F-box*	*EF-1α*	*ACT7*	*PEPKR1*	*ACT7*	*UNK2*	*PP2A*
CV±SD	3.91±1.28	1.60±0.39	2.52±0.82	3.08±0.89	3.14±1.02	2.71±0.79	2.40±0.66
8	*EF-1α*	*PEPKR1*	*TIP41*	*TUA5*	*F-box*	*PEPKR1*	*ACT7*
CV±SD	4.20±1.08	1.61±0.48	2.81±0.75	3.70±0.97	3.16±1.09	2.78±0.84	2.44±0.82
9	*TUA5*	*F-box*	*PEPKR1*	*EF-1α*	*TUA5*	*ACT7*	*TUA5*
CV±SD	4.63±1.31	1.66±0.55	3.34±1.02	4.49±1.08	3.46±1.00	3.65±1.23	2.53±0.67
10	*ACT7*	*ACT7*	*TUA5*	*ACT7*	*PEPKR1*	*TUA5*	*PEPKR1*
CV±SD	4.64±1.62	3.32±1.03	4.07±1.06	5.35±1.73	4.12±1.22	4.39±1.16	2.68±0.83

Notes: SR, roots exposed to high-salt treatment; SL, leaves exposed to high-salt treatment; PR, roots exposed to PEG treatment; PL, leaves exposed to PEG treatment; HL, leaves exposed to heat treatment; CL, leaves exposed to cold treatment. Descriptive statistics of 10 candidate genes based on the coefficient of variance (CV) and standard deviation (SD) of their Cq values were determined using the whole data set. Reference genes were identified as the most stable genes, i.e. those with the lowest coefficient of variance and standard deviation (CV ± SD).

### Reference Gene Validation

**Figure 5:**
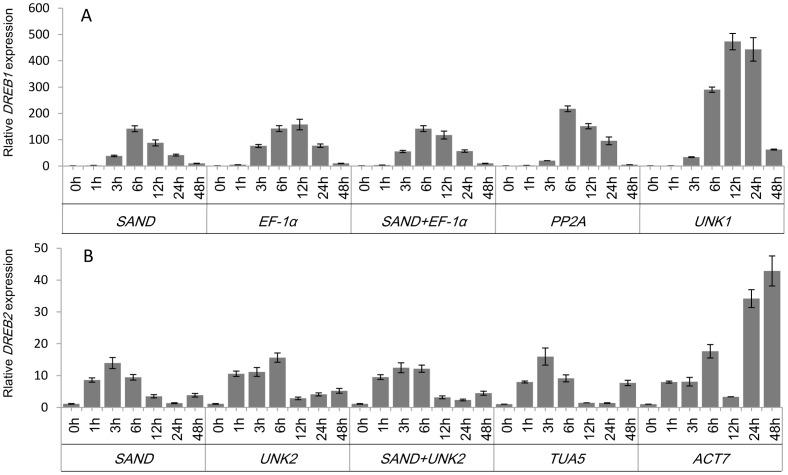
Relative quantification of *DREB1* and *DREB2* expression using validated reference genes for normalization. The results are represented as mean fold changes in relative expression compared to the first sampling stage (0 h). cDNA samples were taken from the same set used for gene expression stability analysis. (A), leaves were collected from three-week-old seedlings subjected to cold stress after 0, 1, 3, 6, 12, 24 and 48 h treatment. (B), roots were collected from three-week-old seedlings subjected to salt stress after 0, 1, 3, 6, 12, 24 and 48 h treatment.

To validate the selection of candidate reference genes, the expression pattern of *DREB1* and *DREB2* were analyzed using the selected reference genes (Figure 5). In *A. thaliana*, expression of the *DREB1* was induced by low-temperature stress, whereas expression of the *DREB2* was induced by drought and high salinity [38]. In this study, expression of *DREB1* in cold-stressed leaves and expression of *DREB2* in salt-stressed roots were assessed.

When the two most stable reference genes, *SAND* and *EF-1α* were used for normalization, the expression levels of *DREB1* increased sharply after 3 h of treatment, peaked at 6 h, and thereafter decreased (Figure 5A). When the least stable gene *UNK1* was used for normalization, the expression patterns and transcript levels were very different. For *DREB2*, when the two most stable reference genes, *UNK2* and *SAND* were used for normalization, the transcript levels of *DREB2* increased rapidly from 1 h, peaked at 3–6 h, and thereafter decreased. The expression level of *DREB*2 peaked at 3 h using just *SAND* and at 6 h using just *UNK2*. Similar expression patterns were generated when the less stable reference gene *TUA5* was employed (Figure 5B). Normalization based on the least stable reference gene *ACT7* found that the transcript level increased rapidly and peaked at 6 h, decreased between 6 and 24 h, and thereafter increased again up to 48 h, which obviously differed from normalization against *SAND* and *UNK2*.

## Discussion

In plant molecular biological research, qPCR has become an important tool for understanding gene expression in different experimental conditions. For accurate qPCR measurements, endogenous reference genes are used as internal controls. An ideal reference gene should be representative of the overall expression across all possible tissues (cells) and experimental conditions [10]. However, such a perfect reference gene is impossible and nothing close has yet been reported. This means that reference genes need to be validated under certain experimental conditions and among various species.

*C. intermedia* is a native desert shrub that is widespread in the desert land of west and northwest China, but the application of qPCR in this species has been limited by a lack of information on reference gene stability in a variety of experimental contexts. Here, we describe the analysis of 10 candidate reference genes to improve relative quantification by qPCR for gene expression analysis in *C. intermedia*. Using three algorithms (geNorm, NormFinder and BestKeeper), we evaluated the expression stability of these ten genes under different abiotic (osmotic, salt, cold and heat) stress conditions in roots and leaves. As far as can be ascertained, this is the first systematic study of the expression stability of reference genes for qPCR in *C. intermedia* under different abiotic stress conditions.

geNorm, NormFinder and BestKeeper are often used to select reference genes. Because they employ different strategies, they can give different results [39], [40]. For example, in the SL treatment, *UNK1* emerged as the most stably expressed using BestKeeper, while it was ranked seventh by geNorm and NormFinder. In the HL treatment, BestKeeper selected *EF-1α* as the most stably expressed gene, but it was ranked sixth by geNorm and seventh by NormFinder. Finally, in the CL treatment, *SAND* and *EF-1α* were identified as the most stably expressed by geNorm, but were ranked third and fifth, respectively, by NormFinder. We considered the results of the three algorithms together when determining suitable reference genes for qPCR normalization (Table S1) [18].

In the SL treatment, the pairwise variation V_3/4_ value (0.1321), calculated by geNorm, suggested the use of *UNK2*, *SAND* and *EF-1α* for normalization. These three genes were also identified by NormFinder. BestKeeper, unlike the other two programs, ranked *UNK1* as most stable (ranked seventh by geNorm and NormFinder), *UNK2* as second, *SAND* as third and *EF-1α* as fourth. Based on these results, we recommended *UNK2* combined with *SAND* and *EF-1α* as the best combination of stable reference genes for qPCR in the SL treatment.

In the HL treatment, the pairwise variation V_3/4_ value (0.148) indicated that the three most stable genes (*SAND*, *PP2A* and *TIP41*) can be used for normalization. BestKeeper ranked *EF-1α* as most stable (ranked seventh by geNorm and NormFinder), *TIP41* as second. NormFinder, ranked *PP2A* as the best reference gene and *SAND* and *TIP41* as third and sixth, respectively. Based on these results, we inferred that *SAND*, *PP2A* and *TIP41* would be appropriate for qPCR in the HL treatment.

In the CL treatment, the pairwise variation V_2/3_ value (0.1282) indicated that the two top ranked genes (*SAND* and *EF-1α*) can be used for normalization. NormFinder ranked *SAND* as third, and *EF-1α* as fifth, however, their stability values did not differ substantially from those of higher-ranked genes (e.g. 0.160 for *SAND* versus 0.142 for first-ranked *PP2A*). BestKeeper ranked *SAND* as most stable, and *EF-1α* as third, similar the results of geNorm. Altogether, we recommended *SAND* and *EF-1α* to be the suitable reference genes for qPCR in the CL treatment. Similarly, *UNK2* and *SAND* would be sufficient for the SR treatment, *PP2A*, *UNK2* and *UNK1* for the PR treatments, and *SAND*, *PP2A* and *TIP41* for the PL treatment.

In summary, *UNK2*, *SAND* and *PP2A* were the most stably expressed genes, while *ACT7* was the most variable, over all samples. *UNK2* has been noted as showing stable expression across tissues and developmental stages in tomato [13], soybean [41] and aspen [42]. *SAND* and *PP2A* have been noted as showing stable expression across tissues and different abiotic and biotic stress conditions in roses [18] and buckwheat [19]. The weakness of *ACT7* was also seen in soybean, where its expression was found to be variable [41].

The transcript levels of *DREB2* peaked at 3–6 h and then began to decline at 12 h in roots under salt stress conditions when *UNK2* and *SAND* were used for normalization (Figure 5B). A similar expression pattern was described under salt stress conditions in *Caragana korshinskii* [43], indicating that the reference genes identified in this study are suitable under such conditions. In the expression profile or transcript abundance quantification produced from normalization using the least stable gene *ACT7*, the transcript level increased rapidly and peaked at 6 h, decreased between 6 and 24 h, and thereafter increased again up to 48 h, which obviously differed from normalization against *SAND* and *UNK2*. Obviously, *ACT7* is not a suitable reference gene to normalize gene expression in *C. intermedia* under such conditions. These results indicate that the incorrect use of reference genes without validation may reduce precision or produce misleading results.

### Conclusions

To our knowledge, this study is the first systematic analysis for the selection of superior reference genes for qPCR in *C. intermedia* under different abiotic (osmotic, salt, cold and heat) stress conditions. Analysis of expression stability using geNorm, NormFinder and BestKeeper revealed that *UNK2*, *PP2A* and *SAND* could be considered to be appropriate reference genes for gene expression analysis of different tissues under different abiotic stress conditions, whereas *ACT7*, *PEPKR1* and *F-box* showed relatively low expression stability. This work will benefit future studies on gene expression under different abiotic stress conditions in *C. intermedia* and other species of the leguminous genus *Caragana*.

## Materials and Methods

### Plant Materials and Treatments

Seeds of *C. intermedia* were collected from the Experimental Base (Hohhot, Inner Mongolia, China), Research Institute of Forestry, Chinese Academy of Forestry. Seeds were washed three times with tap water, and then sown in plastic pots filled with peat soil in a growth chamber with a 16 h light/8 h dark photoperiod at 25/22°C day/night temperatures and relative humidity 80%. For salt and osmotic stress treatments, three-week-old seedlings were carefully removed from the soil to avoid injury, their roots were washed cleanly with tap water, and they were placed in NaCl (200 mM) or PEG6000 (15%) solutions, respectively, for 0, 1, 3, 6, 12, 24, or 48 h in the growth chamber. For the cold and heat stress treatments, the seedlings in pots were grown at 4°C or 42°C, respectively, for 0, 1, 3, 6, 12, 24, or 48 h.

Leaves were collected from the three-week-old seedlings subjected to all four treatments, and roots were collected from the seedlings subjected to salt and osmotic stress treatments. These were immediately frozen in liquid nitrogen and stored at −80°C. Samples above were collected from 3 seedlings to give 3 replicas.

### Total RNA Isolation and cDNA Synthesis

Total RNA was extracted from treated tissues using Trizol reagent (Invitrogen, USA) according to the manufacturer's instructions. The remaining DNA was removed by RNase-free DNase according to the manufacturer’s instructions (Promega, USA). Total RNA concentration and purity was determined using a Nanodrop ND-1000 spectrophotometer (Nanodrop Technologies, USA). RNA samples with an absorbance ratio at OD260/280 between 1.9 and 2.2 and OD260/230 ≈ 2.0 were used for further analysis. RNA integrity was verified by 1.5% agarose gel electrophoresis. Samples with 28S/18S ribosomal RNA between 1.5 and 2.0 and without smears on the agarose gel were used for subsequent experiments.

For each sample, 1 µg of total RNA was reverse transcribed using the RevertAidTM First Strand cDNA Synthesis Kit (Fermentas, Germany) in a 20 µl reaction using oligo dT primers according to manufacturer’s instructions. The cDNAs were diluted 1∶30 with nuclease-free water prior to the qPCR analyses.

### Selection of Candidate Reference Genes

Potential homologues of the ten published reference genes were identified from the transcriptome data sequences of *C. intermedia* seedlings (unpublished data) (Table 1).

The candidate reference genes comprised *ACT7* (actin 7), *EF-1α* (elongation factor -1α), *TUA5* (alpha tubulin), *PP2A* (protein phosphatase2A), *SAND* (SAND-family protein), *TIP41* (TIP41-like protein), *F-box* (F-box/kelch-repeat protein), *PEPKR1* (phosphoenolpyruvate carboxylase-related kinase 1), *UNK1* (hypothetical protein) and *UNK2* (hypothetical protein), which were previously shown to have highly stable expression levels by microarray analysis in *A. thaliana* and soybean [16], [17].

### PCR Primer Design and Test of Amplification Efficiency

Primers were designed using the Primer Premier 5 software (http://www.PremierBiosoft.com/primerdesign/primerdesign.html) with melting temperatures 58–62°C, primer lengths 20–24 bp, GC content 45–55% and amplicon lengths 50–230 bp (Table 1, Table S2 and Figure S1). For each primer pair, amplification efficiency estimates were derived from a standard curve generated from a serial dilution of pooled cDNA (1, 10, 10^2^, 10^3^, 10^4^, 10^5^ × dilutions; each gene in triplicate) (Figure S2). Mean quantification cycle (Cq) values of each ten-fold dilution were plotted against the logarithm of the pooled cDNA dilution factor. Efficiency (E) for each gene was determined with the slope of a linear regression model [44] using the Cq values and the following equation was used:
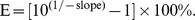



### Quantitative Real-time RT-PCR

qPCR reactions were carried out with an ABI Prism 7700 Sequence Detection System (Applied Biosystems, USA), using SYBR® Premix Ex Taq™ (Takara, Japan) in a 20 µl reaction volume (containing 2 µl diluted cDNA, 10 µl 2 × SYBR Premix Ex Taq™, 0.4 µl ROX Reference Dye, and 0.4 µl each primer). The reaction conditions were: an initial denaturation step of 95°C/30 s, followed by 40 cycles of 95°C/5 s and 60°C/30 s. The dissociation curve was obtained by heating the amplicon from 60 to 95°C (Figure S3). All qPCR reactions were carried out in biological and technical triplicate. A non-template control was also included in each run for each gene. The final quantification cycle (Cq) values were the means of nine values (biological triplicate, each in technical triplicate).

### Statistical Analysis

Three different types of Microsoft Excel-based software, geNorm [45], NormFinder [46] and BestKeeper [47], were used to rank the expression stability of reference genes across all of the experimental sets. Following qPCR data collection, Cq values were converted to relative quantities using the formula: 2^−ΔCq^, in which ΔCq = each corresponding Cq value − minimum Cq value. The sample with the maximum expression level (the minimum Cq value) was used as a calibrator and was set to a value of 1. Relative quantities were used for geNorm and NormFinder, while BestKeeper analyses were based on untransformed Cq values. All three software packages were used according to the manufacturer’s instructions. All other multiple comparisons were performed with SPSS17.0.

## Supporting Information

Figure S1 **qPCR amplification specificity of the 10 reference genes, and** ***DREB1*** **and** ***DREB2*** **.** Amplification fragments were separated by 2% agarose gel electrophoresis.
(PDF)

Figure S2 **Amplification efficiencies of the 10 reference genes, and** ***DREB1*** **and** ***DREB2*** **.**
(PDF)

Figure S3 **Melting curves of the 10 reference genes, and** ***DERB1*** **and** ***DREB2*** **.**
(PDF)

Table S1 The ranking of 10 reference genes by geNorm, NormFinder, and Bestkeeper.
(DOC)

Table S2 List of amplified sequences of the 10 reference genes, and *DREB1* and *DREB2*.
(DOC)
