# Supplementary figures and images for: Reference Gene Selection for Quantitative Real-time PCR Normalization in Caragana intermedia under Different Abiotic Stress Conditions
Source: PLoS One. 2013 Jan 2;8(1):e53196. doi: 10.1371/journal.pone.0053196 (PMC3534648; doi:10.1371/journal.pone.0053196)

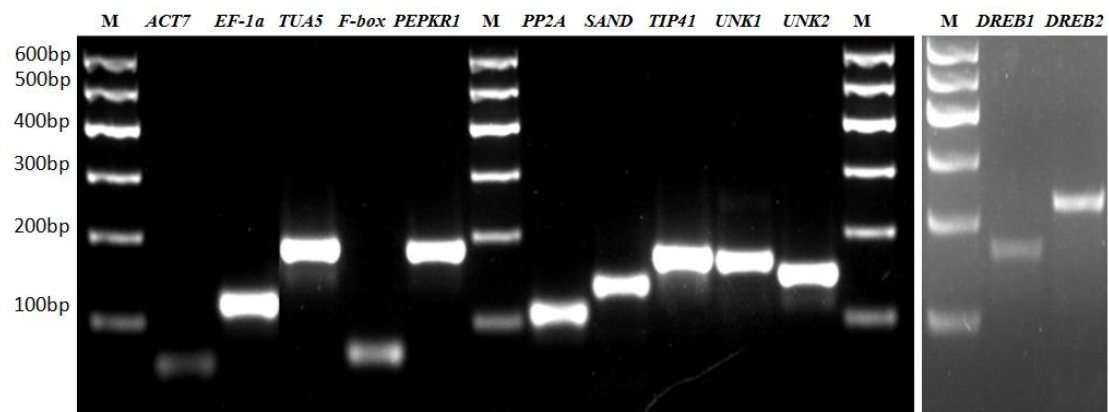

Supplement: Figure S1 — qPCR amplification specificity of the 10 reference genes, and DREB1 and DREB2 . Amplification fragments were separated by 2% agarose gel electrophoresis. (PDF) [file pone.0053196.s001.pdf]

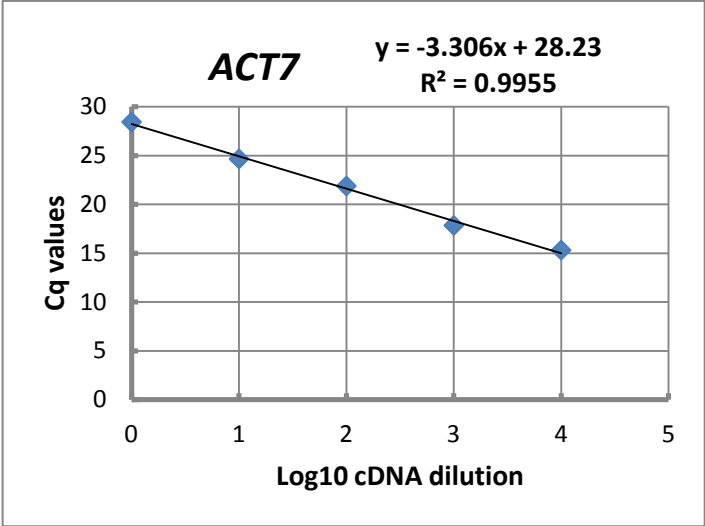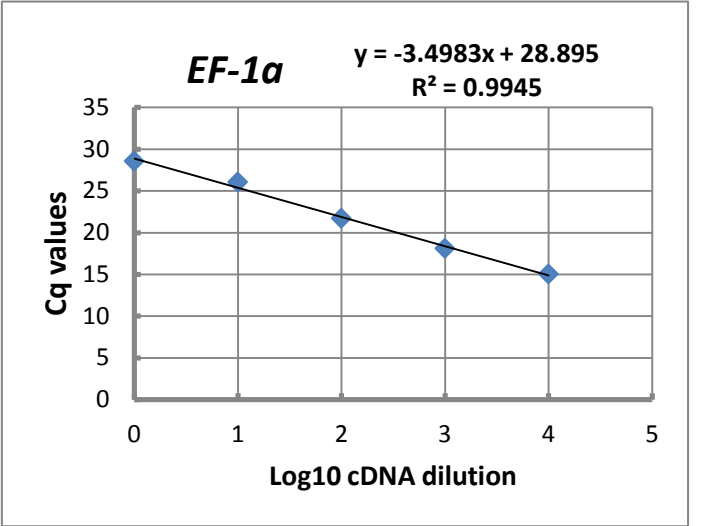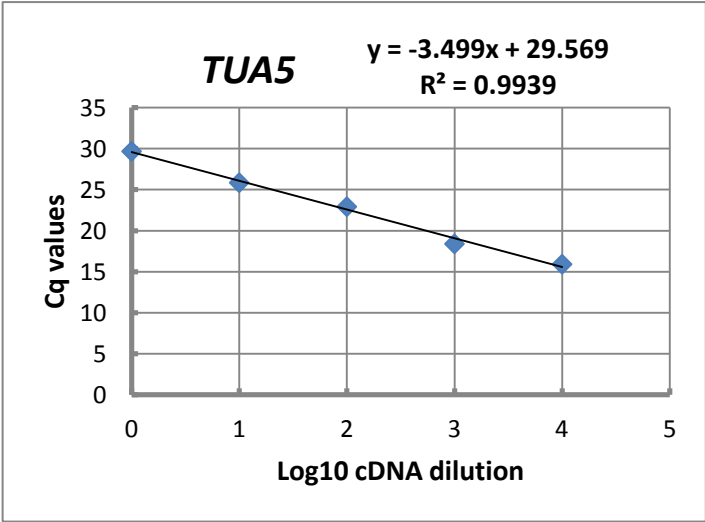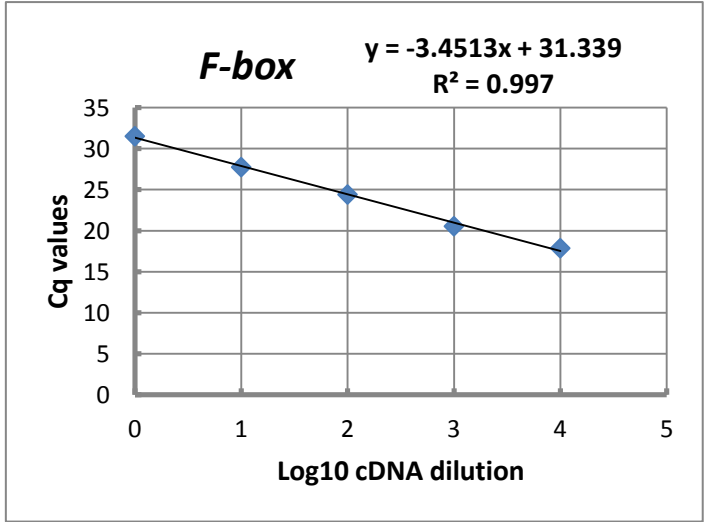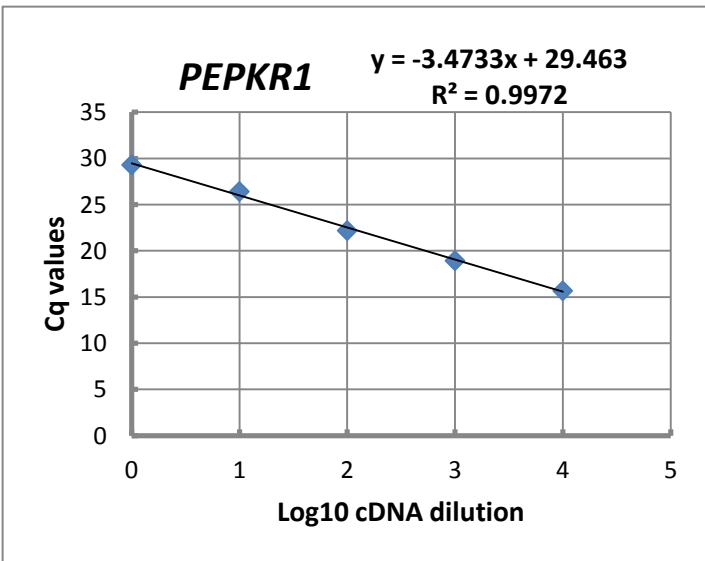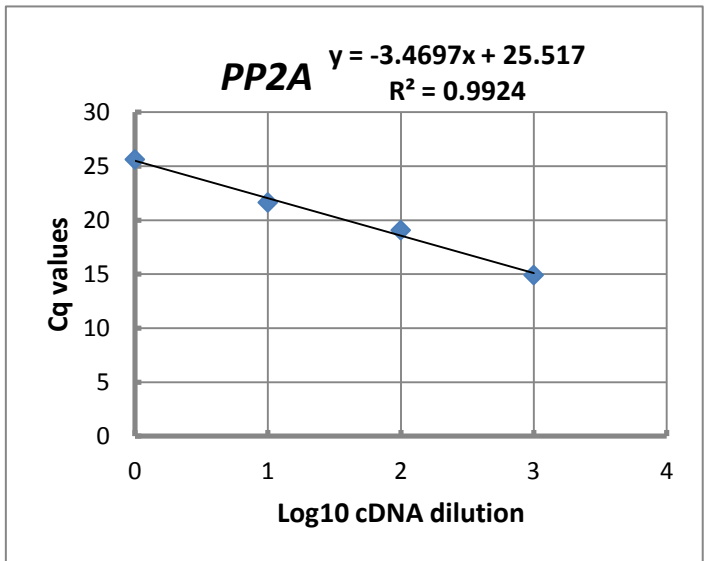

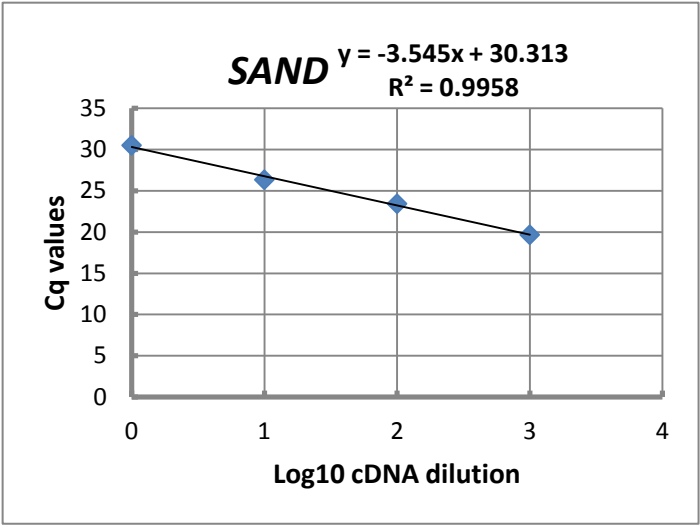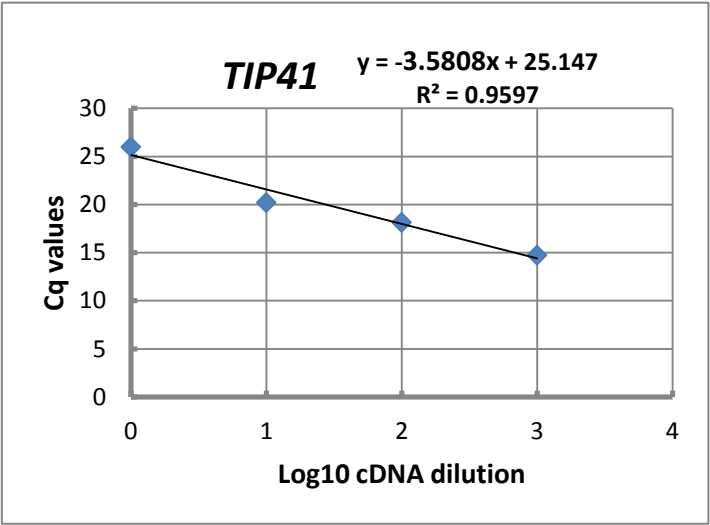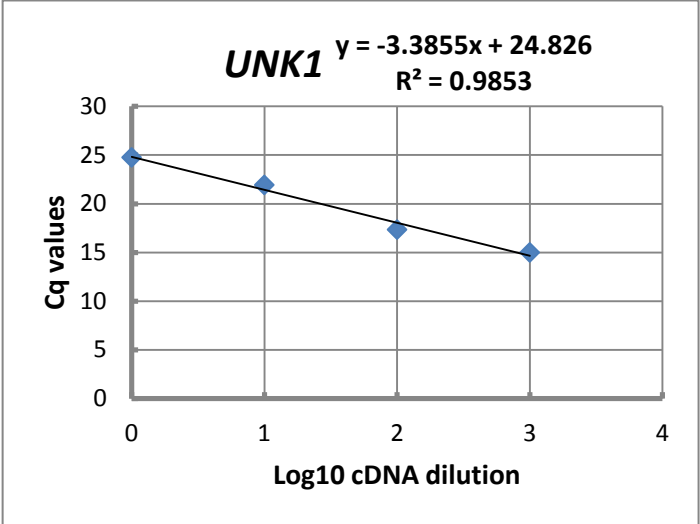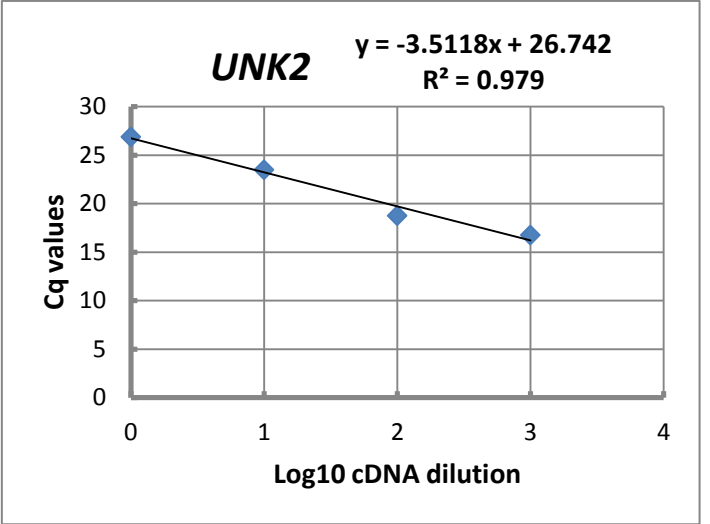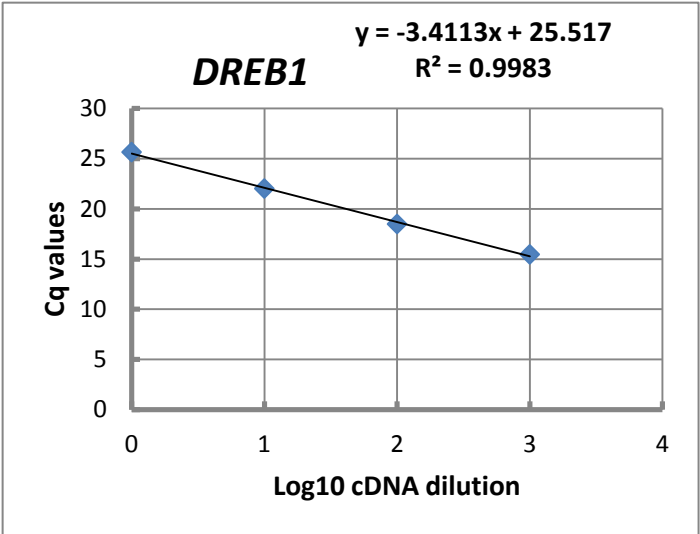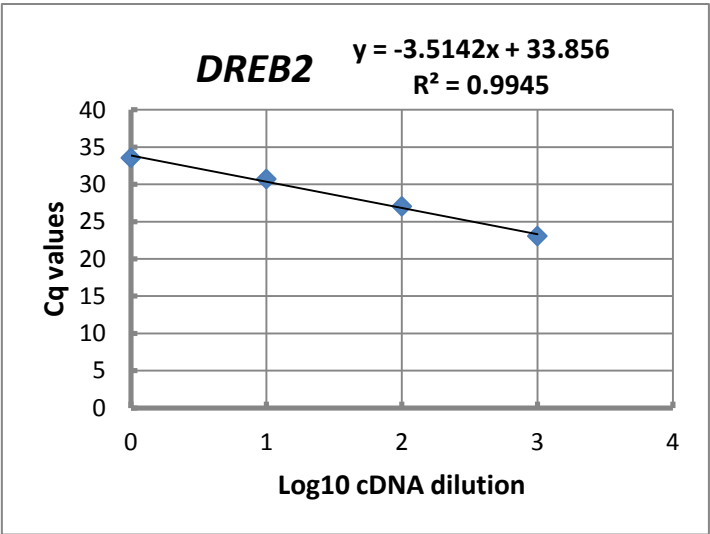

Supplement: Figure S2 — Amplification efficiencies of the 10 reference genes, and DREB1 and DREB2 . (PDF) [file pone.0053196.s002.pdf]

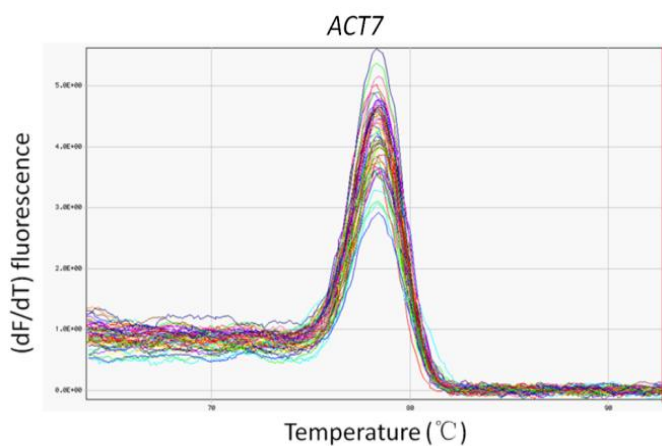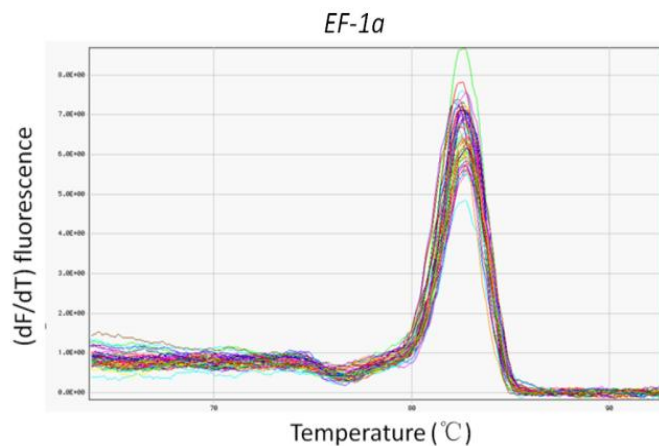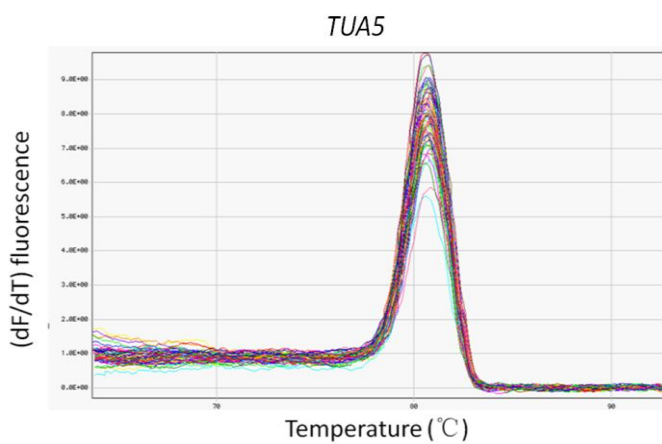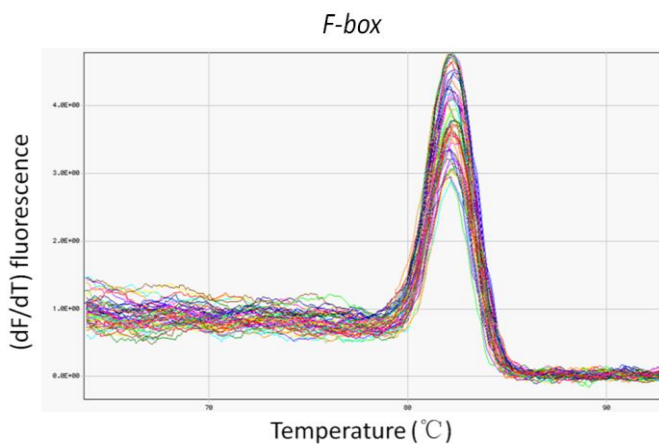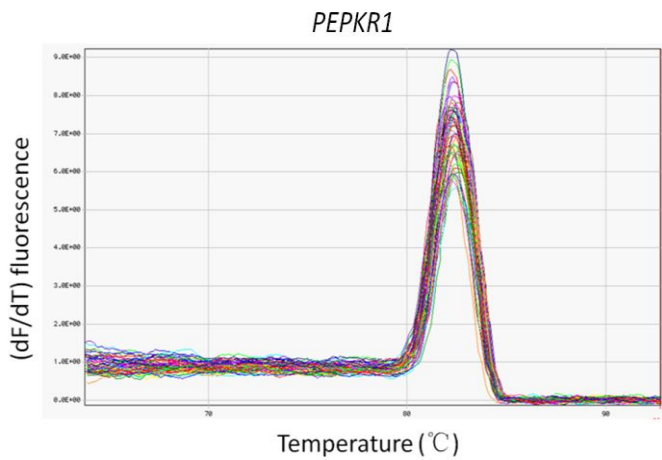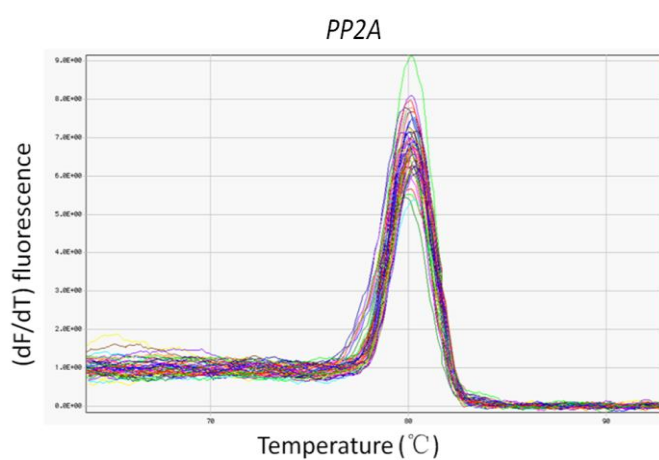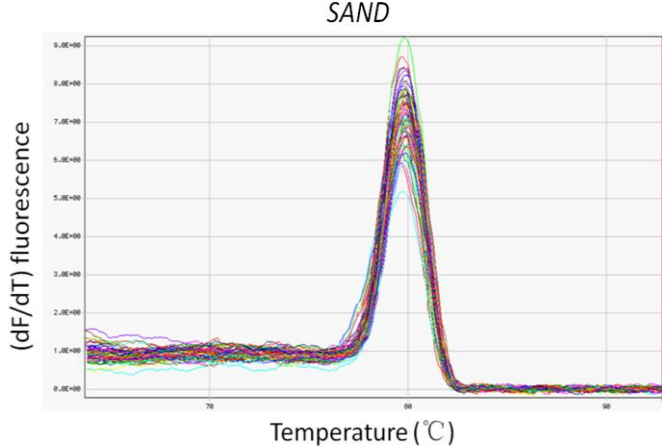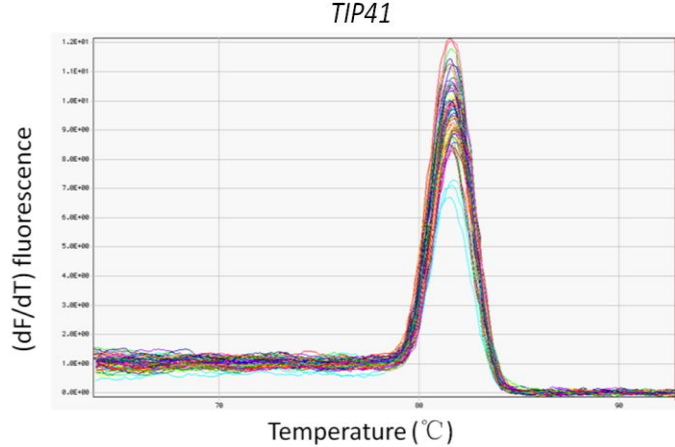

*UNK1*

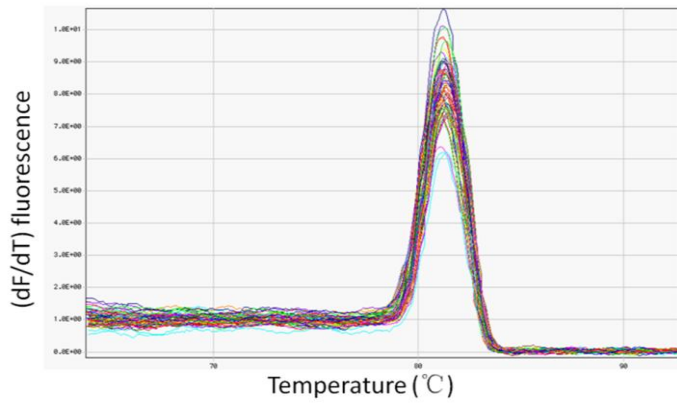

*UNK2*

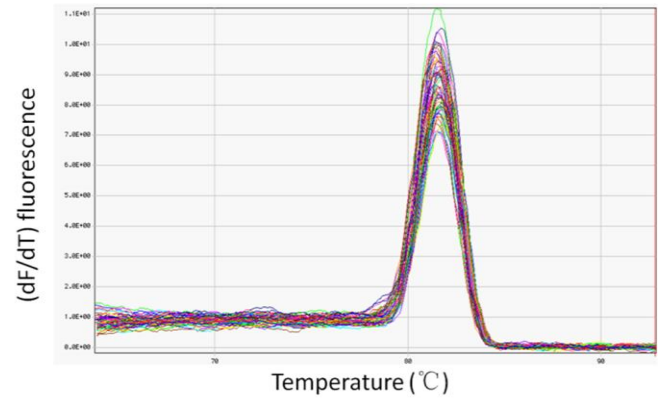

*DREB1*

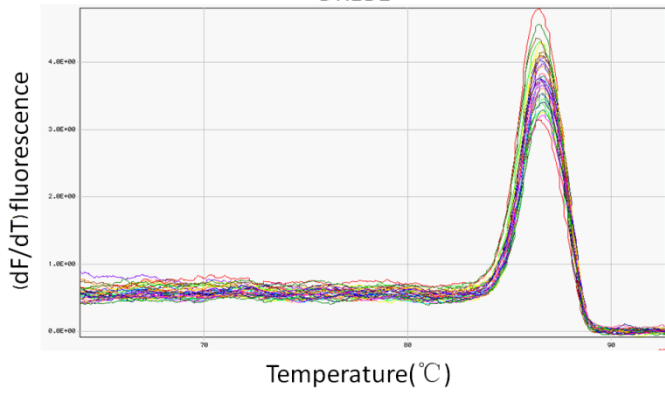

*DREB2*

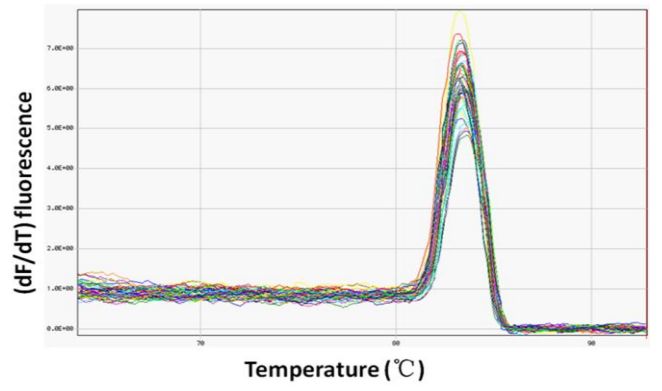

Supplement: Figure S3 — Melting curves of the 10 reference genes, and DERB1 and DREB2 . (PDF) [file pone.0053196.s003.pdf]
