# Supplementary material for: Reference Gene Selection for Quantitative Real-time PCR Normalization in Caragana intermedia under Different Abiotic Stress Conditions
Source: PLoS One. 2013 Jan 2;8(1):e53196. doi: 10.1371/journal.pone.0053196 (PMC3534648; doi:10.1371/journal.pone.0053196)
Supplement: Table S1 — The ranking of 10 reference genes by geNorm, NormFinder, and Bestkeeper. (DOC) [file pone.0053196.s004.doc]

**Table S1. The ranking of 10 reference genes by geNorm, NormFinder, and Bestkeeper**.

| **Rank** | **Software** | **1** | **2** | **3** | **4** | **5** | **6** | **7** | **8** | **9** | **10** |
| --- | --- | --- | --- | --- | --- | --- | --- | --- | --- | --- | --- |
| **Total** | **G** | *UNK2* | *PP2A* | *SAND* | *UNK1* | *TIP41* | *PEPKR1* | *TUA5* | *F-box* | *EF-1α* | *ACT7* |
|  | **N** | *PP2A* | *UNK2* | *TIP41* | *SAND* | *UNK1* | *PEPKR1* | *TUA5* | *F-box* | *EF-1α* | *ACT7* |
|  | **B** | *SAND* | *TIP41* | *PP2A* | *UNK1* | *UNK2* | *PEPKR1* | *F-box* | *EF-1α* | *TUA5* | *ACT7* |
| **SR** | **G** | *UNK2* | *SAND* | *TIP41* | *PP2A* | *TUA5* | *EF-1α* | *UNK1* | *F-box* | *PEPKR1* | *ACT7* |
|  | **N** | *UNK2* | *SAND* | *PP2A* | *TIP41* | *EF-1α* | *F-box* | *TUA5* | *UNK1* | *PEPKR1* | *ACT7* |
|  | **B** | *SAND* | *TIP41* | *UNK2* | *UNK1* | *TUA5* | *PP2A* | *EF-1α* | *PEPKR1* | *F-box* | *ACT7* |
| **SL** | **G** | *UNK2* | *SAND* | *EF-1α* | *ACT7* | *TIP41* | *PP2A* | *UNK1* | *TUA5* | *F-box* | *PEPKR1* |
|  | **N** | *UNK2* | *SAND* | *EF-1α* | *ACT7* | *PP2A* | *TIP41* | *UNK1* | *TUA5* | *F-box* | *PEPKR1* |
|  | **B** | *UNK1* | *UNK2* | *SAND* | *EF-1α* | *F-box* | *PP2A* | *ACT7* | *TIP41* | *PEPKR1* | *TUA5* |
| **PR** | **G** | *UNK2* | *UNK1* | *PP2A* | *SAND* | *TIP41* | *F-box* | *PEPKR1* | *TUA5* | *EF-1α* | *ACT7* |
|  | **N** | *PP2A* | *UNK1* | *UNK2* | *F-box* | *PEPKR1* | *EF-1α* | *TUA5* | *SAND* | *TIP41* | *ACT7* |
|  | **B** | *SAND* | *TIP41* | *UNK1* | *UNK2* | *PP2A* | *F-box* | *PEPKR1* | *TUA5* | *EF-1α* | *ACT7* |
| **PL** | **G** | *TIP41* | *PP2A* | *SAND* | *UNK1* | *UNK2* | *ACT7* | *EF-1α* | *PEPKR1* | *TUA5* | *F-box* |
|  | **N** | *PP2A* | *TIP41* | *UNK2* | *UNK1* | *SAND* | *ACT7* | *EF-1α* | *PEPKR1* | *TUA5* | *F-box* |
|  | **B** | *SAND* | *TIP41* | *UNK1* | *PP2A* | *UNK2* | *EF-1α* | *ACT7* | *F-box* | *TUA5* | *PEPKR1* |
| **HL** | **G** | *SAND* | *PP2A* | *TIP41* | *UNK2* | *PEPKR1* | *EF-1α* | *UNK1* | *F-box* | *TUA5* | *ACT7* |
|  | **N** | *PP2A* | *UNK2* | *SAND* | *UNK1* | *PEPKR1* | *TIP41* | *EF-1α* | *F-box* | *TUA5* | *ACT7* |
|  | **B** | *EF-1α* | *TIP41* | *F-box* | *SAND* | *PP2A* | *UNK1* | *UNK2* | *PEPKR1* | *ACT7* | *TUA5* |
| **CL** | **G** | *SAND* | *EF-1α* | *TIP41* | *UNK2* | *PP2A* | *F-box* | *PEPKR1* | *TUA5* | *ACT7* | *UNK1* |
|  | **N** | *PP2A* | *UNK2* | *SAND* | *TIP41* | *EF-1α* | *F-box* | *PEPKR1* | *ACT7* | *TUA5* | *UNK1* |
|  | **B** | *SAND* | *TIP41* | *EF-1α* | *UNK2* | *UNK1* | *F-box* | *PP2A* | *ACT7* | *TUA5* | *PEPKR1* |

Notes: SR, roots exposed to high-salt treatment; SL, leaves exposed to high-salt treatment; PR, roots exposed to PEG treatment; PL, leaves exposed to PEG treatment; HL, leaves exposed to heat treatment; CL, leaves exposed to cold treatment. 1 represents the most stable gene and 10 represents the least stable gene; G: geNorm, N: NormFinder, B: Bestkeeper.
