# Supplementary material for: Reference Gene Selection for Quantitative Real-time PCR Normalization in Caragana intermedia under Different Abiotic Stress Conditions
Source: PLoS One. 2013 Jan 2;8(1):e53196. doi: 10.1371/journal.pone.0053196 (PMC3534648; doi:10.1371/journal.pone.0053196)
Supplement: Table S2 — List of amplified sequences of the 10 reference genes, and DREB1 and DREB2. (DOC) [file pone.0053196.s005.doc]

**Table S2. List of amplified sequences of the 10 reference genes, and *DREB1* and *DREB2.***

| Gene symbol | Amplified sequences |
| --- | --- |
| *ACT7* | CCGAAGAGCATCCAGTTTTGTTAACAGAGGCTCCTCTTAACCCCAAGGCTAACCGTGA |
| *EF-1*α | AGATGGTTCCCACTAAGCCTATGGTGGGTGGGACATTCTCTGATTACCCTCCTCTTGGTAGGTTTGCTGTGAGGGATATGCGTCAAACTGTGGCAGTTGGAGTCATCAAGAGTGT |
| *TUA5* | CTGATGTGGTTGTGCTTTTGGACAATGAAGCAGTATATGATATATGCAAAAGATCATTAGATATAGAAAGGCCAACTTACACCAACTTGAACCGTTTGATTTCTCAAGTCATCTCTTCCTTGACCACTTCCTTGAGATTTGATGGTGCAATTAACGTTGACATTACCGAGTTCCAGACAAACC |
| *F-box* | AATGGGTCGTGGAGGGTCTAGGAACACAAGCCCTTCCAGGCAGAAGGTGGTTAAGACCAAGCCAAGGGGTT |
| *PEPKR1* | GAACAGTTGGGTTGGGGACAATTTGGTGTCATAAGAGAATGCTCTGACAAGTTGACAGGAGAGGTTTTGGCCTGCAAATCAATTGCTAAAGATAGGTTGGTTACTTTGGATGATTTGAGGAGTGTGAAACTTGAAATCGAGATAATGGCTAGGTTATCCGGGCACCCGAATGTTGTGGATC |
| *PP2A* | TTTCGGATAGGAGGAAATGCACCTGATACCAATTATCTCTTTATGGGCGATTACGTAGATCGTGGTTACTATTCAGTGGAGACTGTTACGCTTTTGGTGGCCTTGA |
| *SAND* | ATACTCGTCAACAGCAGAAAAGACTGTATAAAGCTTACCAGAAACTTTTTGCATCCATGCATGATAAAGGAATCGGGCCACACAAAACTCAATTTAGAAGAGATGAAAACTACGTTCTTTTATGTTGGGTGAC |
| *TIP41* | CGTCCAAGAGTGGGAACAGAAACTTCAGACTTCTCATTTACCAGAAATGGTTTTTGGGGAAAACACTTTAATTCTAAAACACTTGAACAGTGGCACCAAAATTCACTTTAATGCTTTTGATGCTCTCTGTGGCTGGAAACAGGAAGCCTTGCCGCCTGTTGAAGTTC |
| *UNK1* | CAATGTTGAGTGGGGAGGGACAGCTTTATACATACATGGTCCTGATCTCTGAGACAACTACTCCAGGAATCAATTTGAGATGGTATCTCGATGTAGCTGGAATGAAAAGGTCCAAAGCTTATCTCATCAATGGGGTTGTAATATTCCTTGCTTGGCTGGTTG |
| *UNK2* | CAAAGATAGTGCTGCTGATTGCTGTCAAGCTTGCTTGGACCATGCTAAACATGCCAAAGAAGGTGAAAAGAAATGCAATATTTGGGTTTATTGCCCCTCAGAATTTGGATGTCATTCACCAGATATCTATCAGCACAAACACCAGGA |
| *DREB1* | TTCTGACCCACAACCTTACTCCTCAGAGGGGTTTCTTGCAGAGAGCAGGCCGAAGAAGCGTGCAGGGAGGAAGAAGTTCAAGGAGACGCGCCACCCGGTGTACCGTGGTGTGAGGAAGAGGGACTCCGGCAAGTGGGTGTGTGAGGTAAGGGAACCAAACAAGAAGA |
| *DREB2* | GAAAGGGTGTATGAAAGGTAAAGGAGGACCCCAAAACTCAGAGTGTAACTATAGGGGTGTTAGGCAGAGAACATGGGGGAAATGGGTTGGTGAGATTAGAGAGCCAAATAAAGGAAGTAGACTTTGGTTGGGTACTTTTTCTACTGCTCAAGAAGCTGCTCTTGCATATGATGAAGCTGCTAGAGCCATGTATGGTCCCTCTGCACGCCTCAACTTCCCTCACATAAC |
